# Clinical effectiveness and safety of gemifloxacin versus cefpodoxime in acute exacerbation of chronic bronchitis: A randomized, controlled trial

**DOI:** 10.4103/0253-7613.75667

**Published:** 2011-02

**Authors:** S. Chatterjee, T. Biswas, A. Dutta, G. Sengupta, A. Mitra, S. Kundu

**Affiliations:** Department of Pharmacology, Institute of Postgraduate Medical Education and Research, Kolkata, India; 1 Department of Respiratory Medicine, Institute of Postgraduate Medical Education and Research, Kolkata, India

**Keywords:** Acute exacerbation of chronic bronchitis, cefpodoxime, clinical trial, gemifloxacin

## Abstract

**Objective:**

Acute exacerbation of chronic bronchitis (AECB) is a commonly encountered problem and those suspected to be due to bacterial infections require antibiotic therapy. This randomized, controlled trial was designed to evaluate the effectiveness and safety of gemifloxacin, a new fluoroquinolone, versus cefpodoxime, an oral third-generation cephalosporin, for the treatment of mild to moderately severe cases of AECB.

**Materials and Methods:**

Adult subjects diagnosed with chronic bronchitis with clinical symptoms suggestive of an Anthonisen type II acute exacerbation (any two of the following criteria – increased dyspnea, cough, sputum purulence) were eligible and those fulfilling the subject selection criteria were randomized to receive either gemifloxacin 320 mg once daily or cefpodoxime 200 mg twice daily orally for 7 days. The primary outcome measure was clinical success rate at day 14 visit and the secondary outcome measures were changes in Clinical Global impression (CGI) scales and incidence of adverse events (AEs). Fifty-two subjects were enrolled: 26 in gemifloxacin group and 24 in the other and 2 were lost to follow-up.

**Results:**

The clinical success rates were comparable (84.6% in gemifloxacin group versus 83.3% in cefpodoxime group) and no statistically significant difference was observed between the groups. AEs were mild, self-limiting and few (two in gemifloxacin and three in cefpodoxime arm) and tolerability was also good.

**Conclusion:**

The results of this randomized, single-blind trial demonstrated that a 7-day course of gemifloxacin is therapeutically comparable to cefpodoxime in terms of both clinical effectiveness and safety for the treatment of type II Anthonisen category AECB patients.

## Introduction

Acute exacerbation of chronic bronchitis (AECB) is a common but serious respiratory tract ailment associated with significant morbidity and impact on healthcare costs. Bacterial infections are an important cause of AECB. Where bacterial infections are suspected, early institution of antimicrobial therapy and supportive measures ensure quicker recovery.[1–3] The organisms commonly implicated in AECB are *Haemophilus influenzae, Moraxella catarrhalis* and *Streptococcus pneumoniae*, and the less common ones include nonenteric, gram-negative organisms such as *Pseudomonas aeruginosa*.[1,2] The commonly used antibiotics were amoxicillin, trimethoprim, doxycycline which were later on superseded by fluroquinolones, macrolides, second- or third-generation cephalosporins, amoxicillin–clauvulanic acid, due to the emergence of antimicrobial resistance.[4,5] Mild to moderately severe cases of AECB comprise a significant number of patients who attend the outpatient clinics, and institution of oral antibiotic therapy that is effective and tolerable is indicated for those who are of suspected bacterial etiology.[4–6]

Gemifloxacin, a newer generation fluoroquinolone, has pharmacokinetic and pharmacodynamic properties similar to other members of the class but has demonstrated better *in vitro* activity against *S. pneumoniae* with lower minimal inhibitory concentration (MIC) values than the other respiratory fluoroquinolones.[4,5] Antimicrobial activity against gram-negative respiratory pathogens (*H. influenzae, M. catarrhalis*) and atypical pathogens, such as *Chlamydia pneumoniae, Legionella pneumophila*, and *Mycoplasma pneumoniae*, are however comparable.[4,5]

Cefpodoxime, an oral third-generation cephalosporin, has good antimicrobial activity against aerobic gram-positive and gram-negative bacteria like *S. pneumoniae, H. influenzae, M. catarrhalis* and some action against anaerobic organisms as well. There are a few studies which have evaluated the effectiveness of cefpodoxime for various respiratory tract infections and results have shown that it can be considered as an effective agent for the treatment of AECB.[6,7]

*Medline* search revealed comparative clinical trials on gemifloxacin versus other fluoroquinolones or macrolides/amoxicillin–clauvulanic acid in patients of AECB and evaluated its effectiveness both clinically and bacteriologically,[4–7] but till date there has been no comparative study evaluating the efficacy of gemifloxacin versus cefpodoxime for the treatment of the aforesaid disorder in Indian population. Hence, we conducted this randomized clinical trial.

### 

#### Study objectives

The primary objective of this trial was to demonstrate equivalence between gemifloxacin (test) and cefpodoxime (comparator) with respect to their efficacy (clinical treatment success rates) in mild to moderately severe cases of AECB.

The secondary objective was to make a comparative assessment of their safety and tolerability profiles.

## Materials and Methods

This was a prospective, randomized, single-blind, controlled, phase IV (post-marketing) trial. Subject recruitment was done from the Respiratory Medicine outpatient department of the teaching hospital. All study-related documents were approved by the Institutional Ethics Committee and the trial was conducted in accordance with the ICMR guidelines for Biomedical Research on Human Subjects, 2006, and the Declaration of Helsinki.

This clinical trial was designed to demonstrate equivalence in the efficacy (clinical treatment success rates) between the two treatment arms. The largest clinically acceptable effect for which equivalence could be declared was assumed as a difference in clinical cure rates of 10% (equivalence limit). Considering the true mean difference between the treatments as zero and the expected standard deviation of 10% in the trial population, 90% power and α = 0.025, the number of evaluable subjects required in each treatment arm was 26. The sample size was calculated using standard formula for sample size calculation for clinical trials.[8]

### 

#### Inclusion criteria

Adult subjects of either sex, in the age group 25 to 70 years and clinically documented cases of chronic bronchitis, presenting with clinical symptoms and signs suggestive of acute exacerbation of the disease (type II Anthonisen) due to bacterial pathogen, as evidenced by the presence of at least two of the following three *Anthonisen* criteria:[9] exacerbation of cough, dyspnea, and increase in the expectoration volume [increase in the expectoration purulence and a baseline respiratory symptom score (vide Table 1) of >6 but ≤12] were recruited.

**Table 1:** Anthonisen Respiratory Symptom Score

*Signs/symptoms*	*Score 0*	*Score 1*	*Score 2*	*Score 3*
Fever (day time axillary temperature)	<98.6°F	>98.6°F, <100°F,	>100°F but <102°F	>102°F
Increase in cough severity	Nil	Slight	Moderate	Severe
Dyspnea severity	Nil	Slight	Moderate	Severe
Wheeze severity	Nil	Slight	Moderate	Severe
Sputum volume (early morning)	<2 mL	Scanty (3-5 mL)	Moderately copious (6–14 mL)	Copious (>15 mL)
Nature of sputum	Watery	Mucoid	Muco-purulent	Frankly purulent

#### Exclusion criteria

Female patients who were pregnant or lactating or cases of AECB who had severe disease requiring hospitalization or parenteral antibiotic treatment, or suspected or proven cases of pneumonia, bronchial asthma, pulmonary tuberculosis or tubercular pleural effusion, lung cancer or lung metastasis, bronchiectasis, interstitial lung disease or patients who had a course (3 days or more) of antibiotic for respiratory ailments in the preceding 4 weeks of screening or chronic respiratory insufficiency associated with resting hypoxemia or baseline respiratory symptom score <6 and >12 or presence of comorbidities or hypersensitivity to penicillin or any of the study medications were excluded.

Randomization of subjects who fulfilled the selection criteria was done using computerized random number list with recruitment rates in both arms in a 1:1 ratio. Allocation concealment was done by having the random number in an opaque sealed envelope.

Although this was a single-blind trial, where the investigator who clinically assessed the subject was unaware of the treatment allocation and medication, dispensing was done by an independent member who was not part of the study evaluation team.

#### Primary efficacy parameter

Percentage of subjects achieving “treatment success” in each treatment arm was the primary efficacy parameter. Treatment success was defined on the basis of changes in the respiratory scores at day 14 visit and was subdivided as either (a) clinical cure if the respiratory symptom score was <5 at day 14 visit or (b) clinical improvement if the respiratory symptom score was at least one score less than the baseline score or between 6 and 10.

#### Secondary efficacy parameters

Changes in clinical global impression (CGI) scales on a 5-point scale with 1 as the worsened state and 5 as the very much improved state were the secondary efficacy parameters. CGI denoted overall clinical assessment of patient’s condition by the physician and was noted during follow-up visits and at the end of the study. CGI was categorized on a 5-point scale with 1 as the worsened state, 2 as no improvement, 3 as mild improvement, 4 as moderate improvement and 5 as very good improvement state.

Treatment failure was defined as failure to respond to the trial medication and thereby requiring modification of the antibiotic therapy or parenteral antibiotics. A subject was categorized as “treatment failure” if there was no change or increase in the baseline respiratory symptom score at day 14 visit.

#### Study visits and activities

The total duration of the study was 2 weeks; the first 7 days were active treatment period, followed by 7 days of “treatment-free follow-up”. All subjects were evaluated at baseline and at all subsequent follow-up visits (day 3, 7, 14) clinically and the respiratory symptom score (which included symptoms of fever, increase in cough, dyspnea and wheeze severity, sputum volume and nature) was computed. Spirometry, serum creatinine, urea, plasma sugar, hemoglobin, total leukocyte count differential leukocyte count, erythrocyte sedimentation rate (ESR), platelet count and chest X-ray were performed at screening and at study end visit.

#### Study groups

Group A received gemifloxacin tablet (320 mg), while group B received cefpodoxime tablet (200 mg).

Study medications were dispensed once at baseline visit for 3 days and subsequently at the first follow-up visit for the next 4 days. Patients were instructed to take one tablet of their respective study medication (either gemifloxacin 320 mg once daily orally or cefpodoxime 200 mg orally twice daily) after food for the first 7 days. Compliance was assessed by traditional “pill-count” method at each follow-up visit and at the end of the study.

During the study period, subjects were not allowed to take any other systemic antibiotic or indigenous medicines for any medical or surgical cause. However, in cases of treatment failure or worsening of clinical condition, the subject was withdrawn from the trial prematurely. Bronchodilators like beta 2 agonists (salbutamol, levosalbutamol) by inhalational route, anticholinergics, theophylline derivatives and anti-inflammatory agents like inhalational corticosteroids were allowed. All the subjects participating in the trial were advised to stop smoking, and breathing exercises were advised for all subjects during the study period.

Safety monitoring was done continuously throughout the study. All adverse events (AEs) spontaneously reported by the subjects or elicited by the investigators were recorded and causality analysis was done. Any significantly abnormal deviation of baseline investigation report values was considered as an AE and recorded as such.

## Results

Efficacy analysis was done using modified intention-to-treat basis for subjects reporting for at least one post-baseline follow-up visit. All subjects who were randomized were considered for safety analysis. Missing values were dealt with by the last observation carried forward strategy. Parametric tests were used for comparison of normally distributed numerical variables. Categorical data were compared between groups by Chi-squared test or Fisher’s exact test, as appropriate. Repeated measure non-parametric data within group analysis was done by Friedman’s analysis of variance (ANOVA) and between group analysis was done by Mann Whitney U test.

Out of 62 subjects screened, 52 fulfilled the selection criteria and were randomized – 26 to group A (gemifloxacin) arm and 26 to group B (cefpodoxime). However, two subjects were lost to follow-up in group B and did not attend the hospital after the first visit. The protocol specified sample size of 26 “evaluable” subjects could be achieved in group A only within the study period. The mean age of patients was 59.6 ± 6.7 years and 59.2 ± 8.5 years in the gemifloxacin and cefpodoxime groups, respectively. 80.7% were males in the gemifloxacin group and 87.5% were males in the other group. The duration of chronic bronchitis at screening was 7.92 ± 5.02 and 8.95 ± 4.4 years in the gemifloxacin and cefpodoxime groups, respectively.

There was no statistically significant difference in the baseline demographic profile, smoking status and disease related profile (baseline symptom score and duration of chronic bronchitis at screening).

The changes in the respiratory symptom score from baseline values are enlisted in Table 2 and clinical treatment success rates are mentioned in Table 3. Within group analysis of changes in baseline versus first, second follow-up and the study end scores showed a highly significant reduction in both the groups (*P* values included in table), denoting that there was a clinically significant improvement in the signs and symptoms of the acute exacerbation episode of the disease. Therefore, it can be concluded that both gemifloxacin and cefpodoxime are effective antibiotics for the management of AECB. A between group analysis of the symptom scores showed that there was no statistically significant difference in the baseline, first follow-up and end of study scores in the respiratory symptom score. A statistically significant difference was noted in the second follow-up visit (*P* = 0.04). Since in majority of the visits there was no difference, it can be concluded that both gemifloxacin and cefpodoxime are equally effective antibiotics for the management of AECB.

**Table 2:** Within group comparison of respiratory symptom scores

*Visit*	*Group A (gemifloxacin) (n = 26)*	*Group B (cefpodoxime) (n = 24)*
Baseline score		
Mean ± SD	8.9 ± 1.42	9.5 ± 1.17
Median ± IQR	9 ± 2	10 ± 2.7
First follow-up		
Mean ± SD	7.9 ± 1.57	8.7 ± 1.39
Median ± IQR	8 ± 1.5	8.5 ± 1.7
Second follow-up		
Mean ± SD	6.6 ± 2.51	7.5 ± 2.48
Median ± IQR	6 ± 1.5	7 ± 2
End of study		
Mean ± SD	6.5 ± 2.54	7.0 ± 2.62
Median ± IQR	6 ± 0.5	6 ± 2
*P* value	0.0001	0.0001

SD = standard deviation; IQR = interquartile range; Values of P with respect to baseline scores of respective group

**Table 3:** Between group comparison of treatment success rates

*Scores*	*Group A (gemifloxacin) (n = 26)*	*Group B (cefpodoxime) (n = 24)*	*P value*
Treatment success	22 (84.6%)	20 (83.3%)	
Clinical cure	6	3	0.90
Clinical improvement	16	17	
Treatment failure	4 (15.4%)	4 (16.7%)	

Table 3 shows the percentage of subjects who were categorized as “treatment success” or “treatment failure” at the study end visit. From the table, it is evident that 22 subjects out of the 26 enrolled in gemifloxacin group achieved “treatment success,” i.e., either clinical improvement or clinical cure, and the remaining 4 subjects were categorized as treatment failure. Similarly, in the cefpodoxime group, 20 subjects out of the 24 enrolled showed “treatment success,” i.e., either clinical improvement or clinical cure, and the remaining 4 were categorized as treatment failure. Between group comparison of the percentage of subjects who were categorized as treatment success showed no statistically significant difference. The 95% CI of the difference in proportion of treatment success rates between groups was –19.43 to 22.6% which was however outside the assumed equivalence limit of ±10%.

There were four subjects in each group who were categorized as treatment failure and had to be put on other antibiotics after day 7 evaluation. The percentage of treatment failure rates in the two arms was not statistically significant (*P* = 0.90). Changes in CGI assessed by the physician were noted in a 5-point Likert scale at each visit and the results at the study end visit showed that 84.6% of the subjects in gemifloxacin group and 83.3% in cefpodoxime group achieved a score of either 3 or 4 (mild or moderate improvement of the clinical condition). There was no statistically significant difference (*P* = 0.85) of the study end CGI scores in between group comparison. The subject compliance of both the groups was comparable and majority of the subjects showed excellent compliance. There were no subjects who were categorized in the “poor” compliance group.

Safety analysis was done following Intention to Treat (ITT) analysis. Only five AEs were noted during the entire study period - three AEs in cefpodoxime group, which were mild diarrhea, and two AEs in gemifloxacin group, one case each of rash and dizziness. These AEs were non-serious and mild in nature and did not require any dose reduction or withdrawal of the study medications. Causality analysis using the World Health Organization-Uppasala Monitoring Centre (WHO-UMC) criteria[10] showed they were in the “possible” category. Laboratory parameters were within normal ranges in both the study groups and no significant changes were detected between baseline and study end. Therefore, the safety and tolerability profile of both the study drugs were good without any reported cases of serious AE.

The use of concomitant medications in both the groups was comparable and no statistically significant difference was noted between groups. Most of the subjects had taken inhalational salbutamol or levo-salbutamol, ipratropium bromide, and few had taken oral doxophylline or theophyline. There were three diabetics and one hypothyroid patient in gemifloxacin group and two diabetics in cefpodoxime group. However, the percentage of subjects with concomitant illnesses in the two treatment arms was comparable (*P* = 0.75).

## Discussion

The results of this study proved that gemifloxacin is therapeutically comparable to cefpodoxime in clinically suspected cases of AECB, both in terms of efficacy and safety. After treatment with a 7-day course, the clinical success rates were comparable, i.e., 84.6% in the gemifloxacin group and 83.3% in the cefpodoxime group. The number of treatment emergent AEs was also very less, i.e., two in the gemifloxacin group and three in the cefpodoxime group. These adverse events were all mild and non-serious in nature and did not require dose modification or withdrawal of drug therapy. Patient compliance in both the groups was also good.

Several published studies have evaluated the effectiveness of different flouroquinolones (ciprofloxacin, moxifloxacin, gemifloxacin, levofloxacin, prulifloxacin) with macrolides (clarithromycin, azithromycin, roxithromycin) or with amoxicillin/clavulanate acid for the treatment of AECB,[11–16] but there are no published comparative controlled trials which evaluated a fluoroquinolone with an oral third-generation cephalosporin.

The results of this study were comparable to that of other published studies which evaluated gemifloxacin or cefpodoxime for the treatment of AECB using clinical improvement treatment outcomes. The clinical success rate with the newer quinolones was about 83–84%, which is comparable to our study results. Regarding cost effectiveness of a 7-day course of gemifloxacin versus cefpodoxime, we computed the current costs of both therapies by comparing the market prices of the test brand versus the control brand. Results revealed that gemifloxacin therapy was significantly less expensive (about 2.5-fold less Rupees; `100 versus 250) compared to cefpodoxime therapy.

Wilson *et al*. conducted an randomised clinical trial (RCT) of gemifloxacin versus clarithromycin in Anthonisen type I AECB subjects and the reported clinical cure rate at 2-3 weeks was 85.4% with gemifloxacin.[13] Another comparative study of a 5-day course of moxifloxacin versus 7-day course of ceftriaxone showed that the clinical success rates were 90.6 and 89%, respectively.[15] The “Gemifloxacin Long-term Outcome in Bronchitis Evaluation” (GLOBE)[11] trial which compared the clinical and economic outcomes of gemifloxacin versus clarithromycin in AECB patients showed that a 5-day course of gemifloxacin was superior to a 7-day course of clarithromycin and that more patients receiving gemifloxacin remained free of AECB recurrence after 26 weeks compared with those receiving clarithromycin. Very few studies have evaluated the efficacy of cefpodoxime for AECB. A study reported by Periti *et al*.[6] compared the efficacy of cefpodoxime with co-amoxiclav and the results showed that the overall clinical efficacy rate with cefpodoxime was 97.2% versus 94.7% with co-amoxiclav.

Some limitations of this study were as follows. As this was an investigator initiated academic project, a double-blind study could not be conducted due to financial constraints and logistic problems. The dosing schedule of cefpodoxime was twice daily while that of gemifloxacin was once daily. So, for conducting a double-blind study, supply of dummy medications was essential but it could not be arranged. However, to compensate for the bias, the investigator who assessed the subjects clinically and computed the respiratory symptom score was not aware of the subject allocation group. Secondly, we did not perform microbial efficacy of the cases since there are several reports which have stated that mere identification of organisms from the expectorated sputum is not representative of the organism causing AECB as the specimen gets contaminated by the upper airway and laryngeal commensals. Another reason is that very often clinicians start antimicrobial therapy at outpatient setting before the microbial culture report arrives which takes about 72 hours. Therefore, we conducted this study mainly to provide information to clinicians on the comparative effectiveness of these two antibiotics as initial antibiotics for AECB patients based on clinical assessment scores. Third, a prolonged follow-up of subjects to compute the relapse rates also was not done formally as part of the study but we have requested all subjects to come for monthly follow-ups for 6 months after study end visit.

## Conclusion

The results of this study demonstrated that a 7-day course of gemifloxacin is comparable to cefpodoxime in terms of both clinical effectiveness and safety for the treatment of AECB in an outpatient setting. Gemifloxacin has the additional advantage of a once daily dosing compared to a twice daily dosing for cefpodoxime and a lesser cost of therapy. However, a trial that also evaluated bacteriological cure and relapse rates would have provided supportive scientific evidence.
